# Molecular Biomarkers for Embryonic and Adult Neural Stem Cell and Neurogenesis

**DOI:** 10.1155/2015/727542

**Published:** 2015-09-01

**Authors:** Juan Zhang, Jianwei Jiao

**Affiliations:** ^1^State Key Laboratory of Reproductive Biology, Institute of Zoology, Chinese Academy of Sciences, 1 Beichen West Road, Chaoyang District, Beijing 100101, China; ^2^University of Chinese Academy of Sciences, No. 80 Zhongguancun East Road, Chaoyang District, Beijing 100190, China; ^3^Group of Neural Stem Cell and Neurogenesis, Institute of Zoology, Chinese Academy of Sciences, 1 Beichen West Road, Chaoyang District, Beijing 100101, China

## Abstract

The procedure of neurogenesis has made numerous achievements in the past decades, during which various molecular biomarkers have been emerging and have been broadly utilized for the investigation of embryonic and adult neural stem cell (NSC). Nevertheless, there is not a consistent and systematic illustration to depict the functional characteristics of the specific markers expressed in distinct cell types during the different stages of neurogenesis. Here we gathered and generalized a series of NSC biomarkers emerging during the procedures of embryonic and adult neural stem cell, which may be used to identify the subpopulation cells with distinguishing characters in different timeframes of neurogenesis. The identifications of cell patterns will provide applications to the detailed investigations of diverse developmental cell stages and the extents of cell differentiation, which will facilitate the tracing of cell time-course and fate determination of specific cell types and promote the further and literal discoveries of embryonic and adult neurogenesis. Meanwhile, via the utilization of comprehensive applications under the aiding of the systematic knowledge framework, researchers may broaden their insights into the derivation and establishment of novel technologies to analyze the more detailed process of embryogenesis and adult neurogenesis.

## 1. Introduction

Neural stem cells (NSCs) acting as a source of various cell types are a subpopulation of cells that can self-renewal and proliferate identical cells. They are multipotent to generate diversity neural lineages, encompassing neurons, astrocytes, and oligodendrocytes [1]. NSCs serving as an origin of neurons and glia throughout life were one of the milestone events of the past twenty-five years in the neuroscience research field [2], which is quite meaningful to the investigator majoring in the study of NSCs. NSCs with the plasticity to give rise to new neurons and glia play a crucial role in the embryogenesis and adult neurogenesis [3, 4].

The elemental discrimination between embryonic and adult neural stem cells is that the process of adult NSC is not orchestrated and massively paralleled progression as that in the embryonic developmental stages because such stages can occur at any time point [5].

NSCs, a headspring of progenitor cells in the central nervous system (CNS), are born with proliferation capacity of self-renewal and generation of both neurons and glia through a multistep process [6]. During the process of adult neurogenesis, NSCs in the germinal regions undergo numerous stages, including NSCs self-renewal, transient amplifying progenitors, neuroblasts, and terminally mature neurons, astrocytes, and oligodendrocytes [2, 5, 7].

With the various technologies development, a quiet number of molecular biomarkers have been emerging like mushrooms after rain, which will favor the further research in the neuroscience field. However, there is not a systematic framework to illustrate the specific markers' detailed characters and functions. And our summary is tempting to provide such a commentary on these particular cell types for the best use of these powerful cells.

## 2. Molecular Biomarkers during Embryogenesis

**Figure 1:**
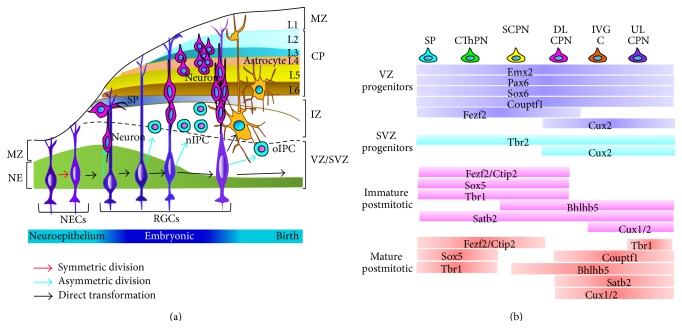
The schematic of embryogenesis and the specific markers expressed in specific time-line. (a) The process of embryogenesis. With the beginning of neuroepithelial cells, a series of cell types are produced, including radial glial cells, neurogenic intermediate progenitor cells, oligogenic intermediate progenitor cells, neurons, and astrocytes. (b) The specific markers indicate the specific cell types generated during the process of neurogenesis [16]. CP, cortical plate; DL, deep layer; GC, glial cells; IZ, intermediate zone; L1–6, layers 1–6; MZ, marginal zone; nIPC, neurogenic intermediate progenitor cell; NECs, neuroepithelium cells; oIPC, oligogenic intermediate progenitor cell; UL, upper layer; CPN, callosal projection neurons. RGCs, radial glial cells; SVZ, subventricular zone; SP, subplate; VZ, ventricular zone. CThPN, corticothalamic projection neurons; SCPN, subcerebral projection neurons.

During the embryogenesis, there are two crucial proliferative zones: ventricular zone (VZ) and subventricular zone (SVZ), which are the springheads of cortical neurons and glia cells [8]. NSCs locate at the VZ of the neural tube and produce all sorts of cell types necessary for the construction of the CNS [9]. The process of embryogenesis can be overviewed in Figure 1.

NSCs in the VZ divide symmetrically and asymmetrically to preserve the stem cell pool and generate progenitor cells, which subsequently migrate to SVZ and then perform the capability of proliferation or differentiation [10]. The SVZ may function as a peculiar zone that instructs the late-born neurons to establish the upper layers and terminally construct the neocortex [11].

The embryogenesis originates from the neural plate which is composed of neuroepithelial cells (NECs). Initially, the NECs divide symmetrically to amplify their own cohorts which are identified as the earliest form of embryonic NSCs [12, 13]. And, after the formation of neural tube, NECs convert to radial glial cells, which locate the soma at the VZ and stretch the long radial fiber out of the neural tube internal surface to the outer (pial) surface [9]. On the one hand, the especial radial glial cells function as a scaffold to guide the migration of neuron. On the other hand, the characterized glial cells present the properties of embryonic NSCs. During this stage, radial glial cells accomplish a process of self-renewal (a newborn radial glial cell) and generate one neuron (or a neuronal progenitor) from each asymmetrical division. Later, radial-glia cells differentiate into ependymal cells forming the neural tube of internal lining, and neurons migrate to the further layers along the radial filaments [14]. At the late stage of embryogenesis, radial glial cells will proliferate to produce oligodendrocytes and eventually astrocytes after the accomplishing of neuron formation. Closing to the date of birth, the radial glial cells change the characteristics to generate NSCs serving as a pool of adult neurogenesis and embryogenesis processes throughout life that can be found at Molecular Biomarkers during Adult Neurogenesis [12, 13].

Given the general frame of embryogenesis, we provide a subsection of this process to get a detailed understanding. The mouse cerebral neocortex can be factitiously partitioned into 6 layers horizontally, each of which contains a specific subpopulation of cells distinguished by singular or multiple markers identifying the characteristics functionally and molecularly [15]. Each layer is composed of pyramidal (glutamatergic excitatory projection) neurons and interneurons (GABAergic inhibitory interneurons). The newborn cortical neurons initially emerge at the mouse gestation of about embryonic day 10.5 (E10.5) and then form the preplate (PP), a cohort of cells located at the surface part (SP) of the cortical mantle.

And at E11.5, the cortical projection neurons present firstly in PP layer and migrate to establish the seminal cortical plate (CP), which whereafter progress to form L2–L6 layers. Before the program of embryonic neurogenesis being launched, the neural progenitor cells (NPCs) in VZ divide symmetrically to amplify the neural progenitor pool. At around E11.5, NPCs get down to divide asymmetrically for self-renewal and to produce neurons which will subsequently migrate to mantle layers (MZ) along the scaffold acted by radial-glia cells (RGCs).

The projection neurons formed at the initial stage locate at the PP and build the nascent CP, which will thenceforth convert into the neocortex L2–6. The increasing CP neurons subsequently crack the PP into SP and MZ. With the neurogenesis progressing, many projection neurons are created in sequence through the continuous asymmetric divisions of NPCs. Gathered together, neurons residing in SP are formed firstly, then those locating at deep layers, and at last those locating at the upper layers (L4, 3, and 2).

The formation of neocortex composed by neurons starts with deep layers; then the newborn neurons will migrate across the older ones to build upper layers. A part of daughter cells of NPCs transform into the IPCs, which will migrate away from VZ and go through symmetric divisions in SVZ, contributing to upper-layer neurons. At around E17.5, closing to the end of neurogenesis, the NPCs convert into gliogenesis, which produce cortical and subependymal zone (SEZ) astrocytes and form the layer of ependymal cells (EL) layer.

During the procedure of embryogenesis, different cell lineages generate different cell types in the different time-courses. And the specific cell types show exclusive cell surface proteins which can be applied to discriminate the particular cell type in specific stage from the NSCs pool. According to the characters above, various cell surface proteins can function as cell markers. And abundant biomarkers have been reported to identify the different cell lineages and different time-courses. Here, we listed the major ones for an illustration.

### 2.1. Emx2

Emx2, empty spiracles homolog 2, belongs to a homeobox-containing gene [18]. It is a transcription factor participating in the development of mouse cerebral cortex including proliferating neuroblasts originating from the neuroepithelium, VZ, and postmitotic Cajal-Retzius cells. Given the enrichment expression at the outset of corticogenesis, Emx2 is used broadly as a dorsal marker during the corticogenesis [19]. The results of Emx2 null embryos indicated that Emx2 as a transcription factor bears a critical part in regulating the neuroblast proliferation, migration, and differentiation. Moreover, Emx2 is also involved in the molding of the forebrain, the definition of cortical territories, and arealization during neocortical development [20].

### 2.2. Sox5

Sox5, sex determining region Y-box 5 (Sox5), is a transcription factor belonging to Sox family. It can be detected exclusively in postmitotic neurons of SP and in projection neurons of L6 at high level and in a cohort of L5 projection neurons. And the detectable phenotype persists from E14.5 to P7 (postnatal 7 days). Meanwhile, Sox5 can also be detected in a few of upper-layer neurons at low levels [21–23].

Otherwise, investigation reported that Sox5 is absent in the progenitor cells residing in VZ and SVZ [23]. In addition, Sox5 is imperative for deep-layer neurons migration across the earlier-born neurons to fix on the more superficial layers. It might manipulate the migration of deep-layer neurons [24].

### 2.3. Bcl11b

Bcl11b (also called Ctip2), B cell leukemia/lymphoma 11B, is a zinc finger transcription factor, which takes part in the development of L5 subcortical axon and is exclusively expressed in L5 [24, 25].

### 2.4. Tbr1

Tbr1, T-box brain factor 1, is a transcription factor, which cooperates with Sox5 to regulate early born neurons in multiple lines during the embryonic development. The deletion of Tbr1 inmice indicated that it is necessary for numerous processes in cortical development, such as laminar location, molecular differentiation, and axonal expeditions [26–28]. The research has reported that Tbr1 could be detected at E12.5 in the corticothalamic projection neurons which located in L6 and SP and in MZ Cajal-Retzius neurons, starting from E12.5 [26]. And, in progenitor cells that reside in VZ and SVZ, Tbr1 is not detectable, which may imply the resemblance of Tbr1 to Sox5 in function. And when time-course progresses to the postnatal course, the layer specificity expression of Tbr1 is gradually downregulated but begins to present in several upper-layer neurons [26, 28, 29]. Additionally, Tbr1 is also involved into the regulation of neuronal migration [27].

### 2.5. Fezf2

Fezf2 (also known as FEZL, ZFP312) belongs to FEZ family zinc finger 2, which also functions as a transcription factor. Fezf2 can be found in L5 cortical spinal (CS) neurons at a high level and plays a pivotal role in the CS tract development. Fezf2 is also found enriching in early progenitors of VZ and in their neuronal progenies which launch into the deep-layer of subcortex. Yet it disappeared in late progenitor cells and upper-layer neurons. Fezf2 is downregulated in L6 neurons during the late embryonic development [27, 28].

### 2.6. Satb2

Satb2, special AT-rich sequence-binding protein 2 (DNA-binding protein), is a matrix-attachment region interacting transcription factor, which can exclusively conjunct the nuclear matrix-attachment regions and plays a role in regulating the transcription and remodelling chromatin. It regulates the position of laminin and helps to identify the late-born neurons. It enriched the postmitotic neurons of corticocortical L2–L5, which begins to emerge in the E13.5 neurons when these neurons migrate into the IZ (intermediate zone) and will persist postnatally. Yet, in the subcortical projection neurons, it is not expressed [30, 31]. Furthermore, SATB2, as an active transcriptional modulator, regulates a diversity of layer-specific markers for cortical projection neurons. The relative markers are listed as follows: Cdh10, cadherin 10; Cux2, cut-like homeobox 2; Rorb, RAR-related orphan receptor beta [31]. Scientists also reported that Satb2 also regulated the neuron dendritic arborization in upper layer (Zhang et al., 2011), referring to a wider role during neocortical development [15, 32].

### 2.7. Cux1/Cux2

Cux1/Cux2, cut-like homeobox 1/2, are upper layer-specific markers for neurons, which participate in the fundamental regulation of the late neuronal differentiation, spine formation, dendritic branching, and synaptogenesis in upper-layer (L2-3) neurons of the cortex [33, 34].

### 2.8. Pou3f2 and Pou3f3

Pou3f2 (POU class 3 homeobox 2, also called Brn-2) and Pou3f3 (POU class 3 homeobox 3, also called Brn-1) both are the members of the class III POU family transcription factors involved in neural differentiation [35]. POU3F2/POU3F3 is considered to be involved in upper-layer neuronal migration and identification, playing overlapping roles in the regulation of neocortical layers development [36, 37]. Pou3f2 and Pou3f3 emerge in the L2–L5 projection neurons at E14.5 and continue presenting in the progenitor cells which amplify themselves and persist running through the migration and differentiation of postmigratory. Studies reported that the absence of Pou3f2/3 led to reduction of upper layer-specific markers expression but did not affect the expression of markers for neurons in L6 and SP [36–38].

### 2.9. Pax6

Pax6, paired box 6, plays a pivotal role in the neuronal fate determination and NSCs proliferation. It participates in the neocortex positioning and upper-layer neurons generation via identifying the SVZ progenitor cells [39, 40]. During the late mouse embryonic developmental stage of the cortical SVZ, Pax6 controls the neural progenitor cells proliferation by changing the Sox2 expression [41].

### 2.10. Nr2f1

Nr2f1 (also known as Coup-tf1), nuclear receptor subfamily 2, group F, member 1, plays a crucial role in the neocortical regionalization. And the late-born neurons migration and the callosal projection neurons (CPNs) differentiation are modulated by Nr2f1 [42, 43].

### 2.11. Sox1

Sox1 is expressed exclusively in the CNS and probably functions as the earliest marker for neural fate decision of embryonic stem cells. Furthermore, it marks the proliferating progenitors residing in the neural tube [44].

## 3. Molecular Biomarkers during Adult Neurogenesis

**Figure 2:**
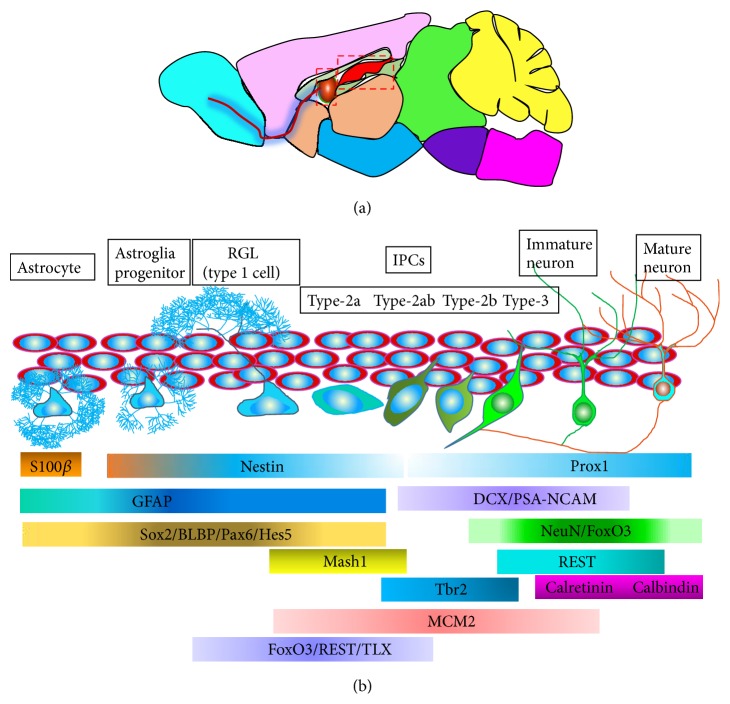
The schematic of adult neurogenesis and specific markers for specific cell types in different time-courses. (a) A model of the two major NSCs niches (labeled by the red panes) in the adult brain. (b) The process of adult neurogenesis originates from the active radial glial cells RGCs, (type-1 cells), generating intermediate progenitor cells (IPCs, type-2a, type-2ab, and type-2b cells), subsequently immature neurons, and finally mature neurons. The specific cell types emerging in the certain strategies are traced by various special markers [17]. GCL, granular cell layer; IPCs, intermediate progenitor cells; SGZ, subgranular zone.

Adult neural stem cells are peculiar cell subpopulations with the character of structural plasticity [17]. In mammals, neurogenesis presents in two germinative regions: SVZ and SGZ throughout life [45]. Currently, various markers expressed at multistep strategies during the progressions of adult hippocampal neurogenesis have been discovered and developed [46, 47], which will be enumerated in the two major procedures of adult neurogenesis. The process of adult neurogenesis can be overviewed in Figure 2.

### 3.1. Developmental Process of Adult Neurogenesis in the Hippocampus

Radial-glia-like neural stem/precursor cells existing in the SGZ are usually regarded as relatively quiescent but can be activated by internal and external stimulus. They compose a pool of neuroblasts including transit-amplifying and proliferative cells produced by symmetrically and asymmetrically dividing. Only a small bunch of cells in this pool can survive and differentiate into immature neurons. After the postdivision of 7–10 days, cells start to enter a neuronal fate [17, 48].

Radial-glia cells act dual-status during CNS development. On the one hand, they play as neural progenitor cells for neuronal generation and a scaffolding facilitating neuronal migration. And on the other hand, they act as a source of most neurons life-long in the CNS [49, 50].

Adult neurogenesis in the hippocampus germinates from progenitor cells and leads to the birth of granule cell neurons, which goes through approximately six distinct stages experiencing type-1 cells, type-2a cells, type-2b cells, type-3 cells and immature and mature neurons [5].

NSCs can be defined as a cohort that possesses both self-renewal and neurons/glia cells production from a unicell according to their potential capacities [1, 51]. NSCs (defined as type-1 cells) amplify intermediate progenitors (IPs, called as Type-2a cells) which keep expressing the stem/progenitor markers involving Sox2, a transcription factor [52]. And, at latish stage, neuronal determination starts to become obvious, overlapping with the transcription factors expression including Prox1, NeuroD1, and doublecortin (DCX) (type-2b) [53]. These cells produce migratory neuroblasts (classified as Type-3 cells) which amplify but subsequently exit the cell cycle ahead of maturation into granule neurons. Presently, the procedure of adult neurogenesis can be separated into six developmental strategies factitiously according to the currently emerging investigations [5, 54].

*Stage 1.* Type-1 cells are a group of radial-glia-like neural stem cells with distinct morphology [55], which express astrocytic marker glial fibrillary acidic protein (GFAP) and nestin [56].

*Stages 2–4.* Type-1 cells assumedly divide asymmetrically generating daughter cells called type-2 cells, which will form subsequently three consistent kinds of putative temporary augmenting progenitor cells, which can be characterized by the proliferation capacities, specific morphology, and their gradually increasing neuronal differentiation [57, 58]. Type-2 cells are GFAP negative and are increasingly capable of proliferation [55, 59]. They enter two subtypes: type-2a, nestin-positive and positive for doublecortin (DCX, an immature neuronal marker); type-2b, nestin and DCX-positive [60]. And type-3 cells, which display the polysialated form of neural cell adhesion molecule (PSA-NCAM), present DCX-positive and nestin-negative features [55, 61]. Meanwhile, the three cell types (type-2a, type-2b, and type-3 cells) share some identical features during the three stages, which are classified into the neuronal lineage and labeled by 5-bromodeoxyuridine (BrdU) [5].

*Stage 5.* After the three stages above, cells are induced eventually to exodus from the cell cycle and enter an ephemeral postmitotic stage entering the early neuronal development and formatting immature neurons which can be marked by DCX, NeuN, and Ca^2+^-binding protein calretinin [62].

*Stage 6*. This is the stage of the ultimately mature new granule cells expressing calbindin. Thereabout 2-3 weeks after the postmitotic stage 5, the new cells transform the calretinin-positive cells to calbindin-positive cells [63].

During the process of neurogenesis, a number of diverse biomarkers can be utilized by immunohistochemical means labeling the specific cell types in diverse states. Following statements will delineate the developmental procedures of neurogenesis with the key identifications, accompanied with generally utilized stage-specific markers.

### 3.2. Molecular Biomarkers in Hippocampal Neurogenesis

With the progress of adult neurogenesis, a series of peculiar cell lineages emerge in turn. Here, we particularize the major biomarkers according to the following capabilities: proliferation, neurogenesis, and gliogenesis.

#### 3.2.1. PCNA

PCNA, proliferating cell nuclear antigen, is important in both the repair and the replication of DNA. The expression of PCNA is increased during the G_1_ and S phases and decreased upon the cell converting into G_2_ and M phases. Nevertheless, this marker can also be detected in the early G_0_ phase, which is caused by the long half-life period of eight to twenty hours [64]. Therefore, it refers to the fact that PCNA is expressed in the whole process of cell cycle. As a proliferation marker, it is usually applied to mark a subgroup of actively dividing cells, which is an indicator of proliferating NSCs (PCNA-positive cells) in SVZ, SGZ, tracing for the elusive adult NSCs [64, 65].

#### 3.2.2. Ki67

Ki67, also known as MKI67, is a nuclear protein, which can be used as a marker for dividing cells. It can be found in all time-courses of cell cycle but G_0_ and early G_1_ phases and the same to quiescent cells [64]. According to the above investigation, both PCNA and Ki-67 can be used to label dividing cells, but PCNA is broader than Ki67 [66].

#### 3.2.3. PH3

PH3, phosphohistone H3, is expressed at the late stage of G_2_ phase and the entire course of M phase during cell division [67]. Given the property of PH3, it is usually applied to seek the cell subgroups in proliferating and mitotic states [68].

#### 3.2.4. BrdU

BrdU (5′-bromo-2′-deoxyuridine), thymidine analog, is usually used to label cells being in the cell cycle of S phase in both embryonic and adult dividing cells. However, BrdU uniquely labeling, without additional markers, is only a prevalent symbolization for neurogenesis. BrdU-positive cells may indicate a subtype of progenitors for new neurons but not progenitors for neuronal cells [69–71].

#### 3.2.5. MCM2

MCM2, minichromosome maintenance protein 2, is associated with the DNA replication. It is expressed at the early stage of G_1_ phase and is sustained throughout cell cycle. Besides, it can be detected in proliferating cells without DNA synthesis too. Its expression level is thus higher than Ki67 which is the short-lived cell proliferation marker [72]. Furthermore, MCM2 has been confirmed to unfold a better tool for the labeling of cell proliferation than Ki67 and has been identified as a more useful marker in adult neurogenesis [73].

#### 3.2.6. GFAP

GFAP, glial fibrillary acidic protein, is an intermediate filament (IF) protein which works as a holder of astrocyte mechanical strength. It is a well-known marker for astrocytes [74]. It has been reported that cells with astrocytic property can serve as an origination of new neurons during adult neurogenesis [75]. Rising proof suggests that GFAP-positive progenitor cells can generate specific cell types of neurons during neurogenesis [76]. Radial astrocytes stretching across the granule cell layer are assumed as potential DG NSCs [55].

#### 3.2.7. BLBP

BLBP, brain lipid binding protein (BLBP), also called B-FABP or FABP7, is subjected to a member of fatty acid-binding proteins (FABPs) family, which are cytoplasmic proteins undertaking fatty acid intake, transportation, and targeting [77]. BLBP extensively serves as a radial-glia cell marker in both embryonic and adult brain developments and is expressed in the astrocyte lineage [78, 79]. It also presents in type-1 cells and a handful of type-2 cells in SGZ [80, 81]. The advent of BLBP in activated astrocytes is related to the expression of oligodendrocyte progenitor cells [50, 82].

#### 3.2.8. Sox2

Sox2, known as SRY (sex determining region Y)box 2, is a member of Sox family of transcription factors. It encodes a highly conserved DNA-binding motif identified as HMG (high-mobility group) box, playing vital roles in distinct stages of mammalian development. Sox2, presenting a high expression in embryonic stem cells and adult NSCs during development [83–85], is a frequency marker for NSCs and is thought to be critical for NSCs proliferation and differentiation. Sox2-positive cells proliferate a subpopulation of undifferentiated, dividing cells in the subgranular zone (SGZ) of adult dentate gyrus. They are capable of generating differentiated cells and identical Sox2-positive cells, indicating their multipotent properties and capacities of self-renewal in the adult brain [86].

On the other hand, Sox1 is also expressed in adult neural progenitor cells and multipotent neural stem cells* in vitro* [87].

#### 3.2.9. PSA-NCAM

PSA-NCAM, polysialylated neural cell adhesion molecule, is highly expressed in neural progenitor cells or mostly glial progenitor cells during brain development [88]. As for the adult brain, PSA-NCAM-positive cells can be focused on the granule cells that are newly generated or in the developing process in the adult brain [89, 90]. It has also been identified as a migrating neuroblasts marker that became neurons in the olfactory bulb (OB) in the SVZ* in vivo* [91]. Additionally, most of PSA-NCAM-positive cells is also NeuroD-positive, doublecortin-positive, NeuN-positive but GFAP-negative. Therefore, PSA-NCAM can be considered as a marker that arrives at the late strategy of adult neurogenesis [89, 90].

#### 3.2.10. NeuroD

NeuroD (neurogenic differentiation) is a transcription factor belonging to the family of basic helix-loop-helix protein. It is presented in later strategies of neuronal progression and is classified as a differentiation indicator for neurogenesis, which may serve as a neuronal determination gene. It can also serve as a specific marker of adult cells in SGZ and inner granule layer [92, 93]. It is basilic for the proliferation of neurons in the adult brain [94]. Meanwhile, the expression of NeuroD can also be detected in PSA-NCAM-positive cells and it is colabeled with about 50% of Pax6-positive cells [95].

#### 3.2.11. Pax6

PAX6, paired box 6, is a member of the paired box (Pax) family. As a conserved transcription factor with different DNA-binding domains (PD, a paired domain, and HD, a paired-type homeodomain), Pax6 is intensely expressed in the cells originating from the embryonic neural development and adult neurogenic niches [96]. It is multifunctional in regulating NSCs proliferation and differentiation by the modulation of various downstream molecules expression. It possesses the capacity of fate specification which sustains the survival of the distinct neuronal subtypes in the adulthood [97]. And its expression in progenitor cells is arrested at the early postnatal periods and is maintained only in adult neural progenitor cells in the restrict regions of SGZ and SVZ [98].

As a transcription factor, Pax6 emerges in embryonic progenitor cells and participates in the cell proliferation and the determination of neuronal fate as a pivotal regulator [99]. Nacher's investigation showed that Pax6 also turns up in the resident cells of adult SVZ and SGZ [95]. In the SGZ, Pax6 can be found in early progenitor cells with radial-glia-like appearance and GFAP/nestin-positive [93, 96]. Yet, there is a small bunch of cells exhibiting both Pax6 and PSA-NCAM or DCX or even NeuroD [100–102]. Pax6, collaborating with Dlx2, is required to specify and maintain neuronal subtype peculiarity in the adult and developing brain [103, 104]. Taking together, Pax6 might be used to classify the newly born cells from the differentiation state in SGZ.

#### 3.2.12. FoxO3

FoxO3 (forkhead box O3) is a transcription factor belonging to the O subclass of the forkhead family which are identified by an evident fork head DNA-binding domain. FoxO factors can lure a series of cellular responses, involving the arrest of cell cycle, cell differentiation, apoptosis, and the opposition to oxidative stress [105]. FoxO3 protein can be detected in adult NSCs/progenitor cells in the adult mouse brain [106]. And FoxO3 probably acts as an activator of apoptosis through upregulating the genes necessary for cell death and downregulating antiapoptotic proteins [107] and as a key player in Notch signaling pathway which is essential for sustaining the adult NSCs quiescent state [108]. The gene expression profile of FoxO3 in NSCs indicates that FoxO3 maintains the homeostasis of mouse NSC pool by provoking the genes that sustains quiescence, prohibits premature differentiation, and regulates oxygen metabolism [106].

#### 3.2.13. Nestin

Nestin, neuroepithelial stem cell protein, is a sort of intermediate filament protein involved in classes VI and IV, which is expressed transiently in adult NSCs, immature neural progenitor cells, and vanishes upon the cells converting into differentiation [109]. It has been frequently utilized as a marker of NSCs both in embryo and in adult brain [110]. Park's data also suggests that nestin is basilic for the survival and self-renewal of NSCs [111].

In the adult mouse brain, nestin-positive cells can be observed extensively in restricted regions, where they might serve as a niche of stem/progenitor cells with the capacity of proliferation and differentiation [112]. During embryogenesis, most nestin-positive cells in early developmental stage indicate a cohort of stem/progenitor cells encompassed in active proliferation [113]. Upon these cells halting to divide and engaging into differentiation, nestin expression will be downregulated [113]. In the adult mouse brain, nestin-positive cells can be observed extensively in restricted regions, where they might serve as a niche of stem/progenitor cells with the capacity of proliferation and differentiation [112].

#### 3.2.14. TLX

TLX, an orphan nuclear receptor (also called NR2E1), is encoded by the tailless (TLX) gene. It can be found in both neural stem/progenitor cells (quiescent NSCs) and transit-amplifying neural progenitors (active NSCs) in the SVZ (subventricular zone) and SGZ (subgranular zone) of adult mouse brain [114–116]. It is important in regulating NSCs self-renewal and proliferation during embryonic development and adulthood via a cell-autonomous mode [117]. And the adult neural stem cell pool consists of TLX-positive cells [116].

TLX is uniquely expressed by astrocyte-like B cells of SVZ. And the deletion of TLX gene may result in an absolute loss of SVZ neurogenesis and the deprivation of NSC property of GFAP-positive cells [115]. Further analysis of TLX indicates an essential role during the identified transition from radial glial cells to astrocyte-like B cells, which suggests that TLX should play as a crucial role in the process of the adult NSCs generation and maintenance in the SVZ [114].

#### 3.2.15. bHLH

The bHLH is a basic helix-loop-helix protein transcription factor family, which regulates vertebrate neurogenesis, showing the capacity of transforming nonneuronal fate to neuronal fate when it is expressed ectopically [118].

#### 3.2.16. Hes5

Hes5 is a member of Hes genes (mammalian homologues of Drosophila hairy and Enhancer of split genes) which can encode a series of basic helix-loop-helix (bHLH) transcriptional repressors. There are three conserved domains (bHLH, Orange, and WRPW domains) involved in Hes5 factor, through which Hes5 factor can set up transcriptional activities [9]. Studies have shown that, during embryogenesis, cells expressing Hes5 are maintained as neural stem cells. Hes5 habituates specifically in the SGZ, being in line with property of progenitor cells in the neurogenic niche [119, 120]. And the Hes5::GFP can mark more restricted and undifferentiated cohorts than Sox2 and BLBP [120].

#### 3.2.17. Mash1

Mash1, mammalian achaete-scute homolog (also called Ascl1), is a bHLH transcription factor, which is essential for embryonic neural differentiation [121]. It is dynamically expressed not only in the intermediate progenitor cells (type C cells) but also in a subpopulation of NSCs potential for long-term neurogenesis in SVZ and SGZ of adult brain [115, 122].

#### 3.2.18. REST

REST, RE1-silencing transcription factor (also known as NRSF), is the GLI-Kruppel class C2H2 zinc finger protein expressed in various neuronal genes [123]. It can silence the gene expression via recruiting mSin3A/B and CoREST [124, 125]. It can be found in nonneural cells and embryonic stem cells at high levels but declines when embryonic stem cells transfer to NSCs and cortical progenitor cells [125]. Conversely, REST highlights obvious levels in granular and pyramidal neurons in adult brain [126]. There is a putative implication that REST shows distinct functions when it arrives at embryonic and adult timelines. The deletion of REST may result in the functional depletion of adult NSC pool, which implies the crucial role of REST in sustaining the quiescent state of adult NSCs [127].

#### 3.2.19. DCX

DCX, doublecortin, a protein facilitating microtubule polymerization, is expressed in migrating neuroblasts and immature neurons, which can be classified as a marker for adult neurogenesis in SGZ. However, not all newly born neurons express DCX. It can be found in newly generated hippocampal, striatal, and olfactory neurons, but not in newly generated neurons in the neocortex [128]. In a word, DCX can be utilized to label the postmitotic neuronal progenitor cells and early immature neurons [60]. And a tiny episode of overlap can be seen between the DCX-positive and nestin-positive cells [129].

#### 3.2.20. Vimentin

Vimentin and GFAP are the two main intermediate filament proteins which imply the property of glial cells. And vimentin is mainly exhibited in the radial-glia and immature astrocytes of the early brain development and vanishes at the terminal of gestation. Simultaneously, GFAP presents in the astroglia cells instead of vimentin. However, Seri's research also suggests that vimentin is expressed in both radial-glia and horizontal cells in SGZ [68, 130].

#### 3.2.21. S100*β*

S100*β*, also called calcium binding protein *β*, is a member of the S100 family, which is anchored at the cytoplasm and nucleus and participates in the procedure of cell cycle and differentiation. It can be detected in a subgroup of specific postmitotic astrocytes [131]. The expression of S100*β* discriminates a cohort of cells losing their NSCs potential from the GFAP-positive cells and indicates a more mature stage [132]. Given the above messages, we can conclude that GFAP^+^ and nestin^+^ progenitor cells are negative for S100*β* [55]. It has been reported that S100*β* could be reflected by immunofluorescent staining in horizontal zone but not radial astrocytes in SGZ [133].

#### 3.2.22. GLAST and GLT1

GLAST (also known as EAAT1) is astrocyte-specific glutamate transporter and GLT1 (also known as EAAT2) is glutamate transporter; both are defined as markers of glial group [134]. GLAST has been found presenting in most of S100*β*-positive cells in SGZ [135]. It initially presents at mouse embryonic days 13/14 (E13/14) and keeps on throughout adulthood [136]. Nevertheless, the results derived from the adult NSCs culture* in vitro* showed that both GLAST and GLT could be detected in neuronal subgroups. Otherwise, investigations also unfolded that nestin-positive and Sox2-positive NSCs could be found expressing GLAST and GFAP [137, 138].

#### 3.2.23. Tbr2

Tbr2, T-box brain gene 2, is a member of the mammalian brain-specific T-box gene family, which was expressed in a couple of regions in the developing brain. Yet, it is absent in most regions of adult brain but hippocampus and OB [139]. Increasing proofs have shown that Tbr2 is involved in adult neurogenesis in SGZ. It is present in a small bunch of Sox2 and Pax6-positive cells but absent in the S100*β*-positive cells. It overlaps with NeuroD-positive cells, as well as DCX^+^ and PSA-NCAM^+^ cells. And Tbr2 gradually disappeared when the cells are predestined to convert into neurons and exit mitotic cycle. Thus, it is not detectable either in calretinin/calbindin-positive immature neurons or in NeuN-positive mature cells. Together, we can conclude that Tbr2 is restrictedly expressed in type-2 and a handful of type-3 progenitor cells [68, 93, 140].

#### 3.2.24. NeuroD

NeuroD, the basic helix-loop-helix protein, is a transcription factor, which has been parted into the group of markers for differentiated cells during neurogenesis in the SGZ [92]. It can be observed in inner granule cell layer and in a half of Pax6 and NeuroD-positive cells as well as PSA-NCAM-positive ones [93, 95, 133]. Gathered together, NeuroD can be defined as a marker for the early neuronal lineage and for the identification of mitotic neuronal cells [68].

#### 3.2.25. Tuj1

Tuj1, neuron-specific class III *β*-tubulin, can be detected in immature neurons starting up at mouse early embryonic 8.5 day and persisting throughout the adulthood. The Tuj1-positive cells can be colabeled with DCX-positive and PSA-NCA-positive cells [141, 142]. Furthermore, it has been reported that Tuj1 can also be detected in basket cells in SGZ [133].

#### 3.2.26. Prox1

Prox1, prosperorelated homeobox gene 1, is necessary for the preservation of IPCs (intermediate progenitor cells) and is needed to promote granule cells to maturate during the procedure of adult neurogenesis [143]. It is absent in nestin^+^ or Sox2^+^ cells but can be found in DCX^+^ cells and calretinin^+^ cells in the adult granule cells [144]. It can be applied to trace the neuronal lineage and mature neurons in the SGZ [53, 144].

#### 3.2.27. CNPase

CNPase, 2′,3′-cyclic-nucleotide 3′-phosphodiesterase, presents specifically in oligodendrocytes in SVZ and SGZ, whose morphology reflected by the immunostaining refers to the beginning of oligodendrocyte differentiation. Furthermore, it is considered to play a leading role in the formation of myelination [145, 146].

### 3.3. Molecular Biomarkers in Adult SVZ

Subventricular zone (SVZ) is the other NSCs pool of the two restricted regions. The SVZ mainly consists of four main cell types: SVZ astrocytes/NSCs (type B cells), intermediate progenitor cells (type C cells), neuroblasts (type A cells), and ependymal cells [75, 147]. The adult SVZ functions as a neurogenesis pool through a surprising resemblance to the embryonic SVZ [148]. Experiencing the embryogenesis, the neonatal radial-glia (RG) cells persist to produce neurons and oligodendrocytes which originate from the intermediate progenitor cells (IPCs). One group of these cells transforms into ependymal cells, and most of the left cells convert into astrocytes (called type B cells) locating at adult SVZ, which continue to serve as NSCs throughout the adulthood. B cells preserve the epithelial formation which fix the apical into the ventricle zone and terminate the basal layer in blood vessels. Type B cells express markers tracing for astroglia, such as GFAP, glial fibrillary acidic protein; GLAST, astrocyte-specific glutamate transporter; BLBP, brain-lipid-binding protein; and nestin [148, 149].

And the slowly dividing astrocytes (B cells) continue to produce precursor C cells (intermediate progenitor cells), and C cells can produce the specific lineage A cells (neuroblasts) which will migrate along the RMS (rostral migratory stream) to the OB (olfactory bulb) where they finish their destined mission and convert into granule neurons [150].

GFAP specifically marks the NSCs and astrocytes residing in the SVZ but does not mark the subgroup of TAPs (transient amplifying progenitor cells) [78]. Meanwhile, Ki67, MCM2 (minichromosome maintenance 2), and PH3 (phosphohistone H3) can be used to mark the nuclei of proliferating cells, which make NSC nuclei visual in single one.

Sox2 marks the active astrocytes (NSCs) and neural progenitors (TAPs) of SVZ [75, 84]. PSA-NCAM marks the neuroblasts. NG2 and Mash1 exclusively mark the TAPs and a subgroup of migrating neuroblasts [151]. S100*β*marks the astrocytes and ependymal cells [152]. CD24 marks the migrating immature neurons and ependymal cells. CD31 (PECAM-1) marks the vascular endothelial cells [153]. In the SVZ, cells that transform into another morphology through a series of time-courses can be marked by Tuj1 for neurons, GFAP for astrocytes, and O4/CNPase for oligodendrocytes (O4 and CNPase) [154].

Besides the markers mentioned above, a number of biomarkers reported in abundant investigations are remaining. Here, we enumerate some of them that were not illustrated in this review.

*Astrocytes Markers.* These include Aldh1L1, aldehyde dehydrogenase family 1 member L1; Bysl, bystin; GjA1, connexin 43; Glul, glutamine synthetase; PygB, glycogen phosphorylase; Slc1A2 GLT-1, excitatory amino acid transporter 2; Slc1A3, Glast-1, excitatory amino acid transporter 1. 

*Radial-Glia Markers.* These include Aqp4, aquaporin-4; Slc1A2 GLT-1, excitatory amino acid transporter 2. 

*Oligodendrocyte Markers.* These include Olig2, oligodendrocyte transcription factor; Car2, carbonic anhydrase 2; NFIA, nuclear factor 1 A-type; NFIB, nuclear factor 1 B-type; NFIX, nuclear factor 1 X-type; NSCs: NFIA, nuclear factor 1 A-type; NFIB, nuclear factor 1 B-type; Slc1A2 GLT-1, excitatory amino acid transporter 2.

## 4. Concluding Remarks

Given so abundant investigations and reviews by so many prominent scientists, we can now sketch the outline of various biomarker functional characteristics and latent application in multiple aspects. Gathering all of the formulations above, we catch a sight of many milestones established in the development of neurogenesis as well as obstacles being unfathomed.

Neural stem cells (NSCs) are the fundamental source of all cell types in the CNS. To grasp the nature of niche components in the mouse brain, we need to understand the major characteristics and functions of each element in the niche, to comprehend what role each cellular member plays in the procedure of NSCs maintenance, proliferation, and differentiation. However, it is difficult, using a single marker, to identify a single cell lineage exclusively. And thus it is necessary to apply multiple markers to the analysis of one peculiar cell type. Recently, numerous methods and technologies have been emerging to classify and identify the specific cell types during the neurogenesis of both embryonic and adult brains. Nevertheless, each approach possesses shining points and flaws. We need more concerns to consummate them.
